# Blue spaces and incident dementia: Differences by geospatial and historical contexts

**DOI:** 10.1002/alz.70850

**Published:** 2025-10-28

**Authors:** Kyle D. Moored, Michael R. Desjardins, Andrea L. Rosso, Gina S. Lovasi, Timothy M. Shields, Frank C. Curriero, Oscar L. Lopez, Michelle C. Carlson

**Affiliations:** ^1^ Department of Mental Health Johns Hopkins Bloomberg School of Public Health Baltimore Maryland USA; ^2^ Department of Epidemiology and Spatial Science for Public Health Center Johns Hopkins Bloomberg School of Public Health Baltimore Maryland USA; ^3^ Department of Oncology and The Sidney Kimmel Comprehensive Cancer Center Johns Hopkins School of Medicine Baltimore Maryland USA; ^4^ Department of Epidemiology University of Pittsburgh School of Public Health Pittsburgh Pennsylvania USA; ^5^ Department of Epidemiology and Biostatistics Drexel University Dornsife School of Public Health Philadelphia Pennsylvania USA; ^6^ Departments of Neurology and Psychiatry University of Pittsburgh Pittsburgh Pennsylvania USA

**Keywords:** aging, Alzheimer's disease, built environment and health, geographic information system (GIS), urban planning

## Abstract

**INTRODUCTION:**

Blue spaces (i.e., water bodies) may benefit cognitive health depending on their uses and surrounding spatial context. We examined associations between blue spaces and incident dementia in the Cardiovascular Health Cognition Study, and specifically within Pittsburgh, given its industrial uses of blue spaces.

**METHODS:**

Participants were 2924 adults (Pittsburgh: *n* = 651) ≥65 years of age. Dementia was clinically adjudicated (1992–1999). Water density was measured using both 1 km radial buffers and U.S. Census tracts.

**RESULTS:**

In Pittsburgh only, greater buffer‐level blue space predicted a higher risk of mixed/vascular dementia (highest vs lowest tertile: hazard ratio [HR] = 2.87, 95% confidence interval [CI]: 1.43–5.74), but not Alzheimer's disease (*p* > 0.05). This was attenuated adjusting for individual/neighborhood confounders (HR = 2.65, 95% CI: 0.99–7.07). Tract‐level associations were attenuated but significant after adjustment.

**DISCUSSION:**

Blue space was related to vascular dementia risk after accounting for social context and using more personalized buffer‐level measures. Future studies should carefully consider spatial units and differentiate blue spaces by historical uses.

**Highlights:**

- Examined associations between nearby blue spaces and incident dementia.
- Tested multiple spatial units (buffer, tract) and separately for the Pittsburgh site.
- Blue space density was not related to dementia risk in overall sample.
- Greater blue space density predicted a higher risk of dementia for the Pittsburgh site.
- Associations were attenuated after adjusting for neighborhood confounders.

## 1 BACKGROUND

The growing availability of geospatial data has fueled a rapid expansion of epidemiologic studies exploring how natural environmental features influence health during aging. This is especially true for studies of cognitive aging and Alzheimer's disease and related dementias (ADRD), for which treatments are currently limited but behavior‐based prevention strategies show promise (e.g., promoting physical activity, reducing vascular risk).^1^ Several studies have linked environmental features, such as green spaces and walkable facilities (e.g., recreation centers, civic/social organizations), with reduced ADRD biomarkers in older adulthood.^2^, ^3^, ^4^ These features offer promising targets for structural interventions on ADRD.

One underexplored environmental contributor to cognitive aging is blue space. Blue spaces are outdoor areas with surface water bodies (e.g., lakes, oceans) or watercourses (e.g., rivers) that are accessible to humans.^5^ The few existing studies on this topic have largely found null associations between the quantity of neighborhood blue space and risk of ADRD.^6^, ^7^ One potential reason for this is that proximity to water has a complex relationship with cognitive health.^8^ Physiologically, recreational blue spaces may encourage physical and social activity that promotes cardiovascular health, builds brain reserves (e.g., via neurotrophic factors), and reduces allostatic load, all of which have been linked with improved cognition.^1^, ^9^, ^10^, ^11^, ^12^, ^13^ Psychosocially, blue spaces may lower stress, anxiety, and depressive symptoms that increase the risk of ADRD.^8^, ^12^, ^14^ In contrast, blue spaces used primarily for industrial shipping may provide fewer recreational opportunities and further expose individuals to air and noise pollution that increase risk of cognitive decline.^15^, ^16^ Ultimately, the complexity of the relationship between blue spaces and cognitive functioning requires characterizing not only the availability of blue spaces but also the context surrounding these spaces. Yet, a common issue in the existing literature is a lack of consideration for how geospatial, historical, and social contexts shape the built environments in which we spend time, including those surrounding blue spaces.

Here we demonstrate how ignoring geospatial, historical, and social contexts may lead to potentially incorrect conclusions about how blue spaces contribute to cognitive aging. We examined this in the Cardiovascular Health Cognition Study (CHCS), a prospective cohort study of U.S. older adults enrolled between 1989 and 1993. We evaluated contextual influences in two ways. First, studies of built environmental features typically use a single spatial unit, oftentimes U.S. Census boundaries, which may not overlap with where the participant was active and ultimately most exposed to environmental factors.^17^ This spatial misalignment can lead to biased estimates of associations between blue spaces and cognitive outcomes. We therefore used multiple spatial units to define blue space.

Second, to illustrate the importance of historical uses of blue spaces, we also separately examined participants from the Pittsburgh, Pennsylvania site of CHCS. We compared these findings with those from the entire CHCS sample aggregated across geographic regions (i.e., study sites), which may mask region‐specific patterns important to understanding how local historical contexts may influence ADRD risk. Unlike other CHCS sites, Pittsburgh was a major U.S. steel producer in the early 20th century up until the decline of the industry in the 1980s.^18^ Many steel mills were located along the city's major rivers, and the transport of steel occurred primarily by water or railway.^19^ Collectively, industrial activity along waterways potentially exposed nearby CHCS participants to higher levels of air and noise pollution that may harm cognitive health.^15^, ^16^, ^20^, ^21^ Even today, areas around the city's major rivers have higher concentrations of air pollutants linked with higher ADRD risk.^22^, ^23^

We therefore hypothesized that having a greater density of surrounding water at baseline would be associated with a greater risk of ADRD for Pittsburgh participants, but not necessarily all participants, in CHCS. We further hypothesized that this relationship would be attenuated after adjusting for environmental measures of socioeconomic status or industrialization, suggesting the importance of considering these confounding contextual influences when assessing how blue spaces may affect cognitive health. Finally, we explored whether blue space may act through specific pathological pathways by performing separate analyses by dementia subtypes (Alzheimer's, mixed/vascular).

## 2 METHODS

### 2.1 Study sample

CHCS included 3608 participants across four study sites: Hagerstown, Maryland; Pittsburgh, Pennsylvania; Sacramento, California, and Winston‐Salem, North Carolina.^24^ To be eligible, participants needed to complete cranial magnetic resonance imaging (MRI) and a Modified Mini‐Mental State Examination (3MS) between 1992 and 1994. Participants with prevalent dementia (*n* = 227), missing covariates (*n* = 64), or who were ineligible for geocoding (*n* = 393) were excluded, resulting in an analytic sample of 2924. In addition to the full CHCS analytic sample, we also separately examined participants at the Pittsburgh site who resided in Allegheny County, Pennsylvania (*n* = 651). All participants provided informed consent, and study protocols were approved by the institutional review boards at the University of Washington Coordinating Center and each individual study site.

**RESEARCH IN CONTEXT**

1. **Systematic review**: The authors searched online sources (e.g., PubMed) for related literature using terms such as “neighborhood,” “blue space,” “open water,” “dementia,” and “Alzheimer's disease.”
2. **Interpretation**: Greater neighborhood blue space density was associated with greater risk of incident dementia in Pittsburgh, especially dementia with vascular or mixed vascular/Alzheimer's pathologies. This association was attenuated after adjusting for additional neighborhood confounders, including socioeconomic status and railway density, which accounted for how many areas with major blue spaces in Pittsburgh were historically industrialized and working‐class neighborhoods. Associations were further attenuated using more personalized buffer‐level vs tract‐level measures, suggesting that any observed relationship with blue space may be driven in part by the selected spatial unit.
3. **Future directions**: Studies should (1) better distinguish types of blue spaces (e.g., recreational vs non‐recreational) that may differentially affect cognitive health using additional historical and contemporary environmental measures, and (2) incorporate sensitivity analyses to assess the robustness of findings across spatial scales.

### 2.2 Measures

We measured blue space density in 1992 (baseline) as the proportion of open water from the National Land Cover Dataset.^25^ Two spatial units were used: (1) U.S. Census tracts and (2) 1 km radial Euclidean buffers around the participant's home. The 1 km buffers were thought to capture exposures centered around the participant's home and therefore be more spatially aligned than tract‐level measures, for which the participant's home may be near the boundary rather than in the center. We also generated a tract‐level neighborhood socioeconomic status (nSES) index using four Census measures from the 1990 Longitudinal Tract Database: (1) median home value, (2) median household income, (3) percentage of residents with at least a 4‐year college degree, and (4) percentage of residents with a professional/managerial occupation.^2^, ^26^, ^27^ For the Pittsburgh site, we quantified tract‐level railway density using 1992 Census TIGER/Line data as a proxy for industrial exposures, given the high use of industrial railway shipping in this region.^19^ Railway density was calculated as total railway length divided by tract‐level area (km/km^2^). All neighborhood measures (blue space, nSES, railway) were stratified into approximate tertiles for analysis (low, middle, high). Given that blue space and railway measures were highly positively skewed (i.e., most participants did not live near blue spaces), the highest category instead represents approximately the top decile of these measures.

Time to incident dementia in CHCS was assessed as the number of years from baseline MRI (1992–1994) to the earliest of onset of dementia, death, or end of dementia follow‐up (June 1999).^24^, ^28^ Median follow‐up was 6.0 years (interquartile range [IQR]: 4.6, 6.5). Cases were adjudicated using multiple data sources (e.g., neuropsychological measures, neurological exam, and medical records) by a clinical committee representing each study site.^24^ Dementia subtypes were classified after review of the baseline MRI using established diagnostic criteria: Diagnostic and Statistical Manual of Mental Disorders, Fourth Edition (DSM‐IV),^29^ the National Institute of Neurological and Communicative Disorders and Stroke–Alzheimer's Disease and Related Disorders Association (NINCDS‐ADRDA; Alzheimer's subtype),^30^ the National Institute of Neurological Diseases and Stroke – Association Internationale pour la Recherche et l'Enseignement en Neurosciences (NINCDS‐AIREN, vascular subtype),^31^ and State of California Alzheimer's Disease Diagnostic and Treatment Centers (ADDTC, vascular subtype).^32^ Cases generally had deficits in at least two cognitive domains and showed progressive or static cognitive impairments that were sufficiently severe to impact daily functioning.^28^

Individual‐level participant demographic, health, and socioeconomic characteristics were assessed at study entry. Participants self‐reported their age, sex, race, education, marital status (married, widowed, divorced/separated, never married), current income (<$12k, [$12–25k), [$25–35k), ≥$35k, missing/refused), and primary lifetime occupation (professional/technical/managerial/administrative, sales/clerical service, craftsman/machine operator/laborer, farming/forestry, housewife, other, refused to answer). Physical activity was measured as weekly energy expenditure (kcal/week) from self‐reported leisure and household activities on the Minnesota Leisure Time Questionnaire.^33^ Depressive symptoms were assessed using the total score on the 10‐item Centers for Epidemiologic Studies Depression Scale (CES‐D).^34^ Multimorbidity was assessed by summing the total number of the following medical conditions (range: 0–4): hypertension (systolic blood pressure *>*140 mmHg, diastolic blood pressure *>*90 mmHg, or medication use), diabetes (fasting glucose *>*125 mg/dL, non‐fasting ≥200 mg/dL, or medication use), cardiovascular disease (coronary heart disease, myocardial infarction, congestive heart failure), and cerebrovascular disease (stroke, transient ischemic attack).

### 2.3 Statistical analysis

Descriptive statistics were examined for all measures for the overall sample and stratified by blue space tertile. We used analyses of variance with post hoc pairwise comparisons (continuous variables) and chi‐square tests (categorical variables) to test differences between blue space tertiles. We fit cause‐specific Cox proportional hazards models of time to dementia, treating death as a censoring event, which has been recommended in the literature and found to yield more biologically plausible risk factor‐outcome associations than alternative competing‐risk approaches (i.e., Fine and Gray).^35^, ^36^ Models were adjusted sequentially for baseline covariates. Model 1 was age‐adjusted. Model 2 was further adjusted for individual sociodemographic and health factors (sex, race, clinic site, education, income, lifetime occupation, depressive symptoms, physical activity, and number of medical conditions). Model 3 was further adjusted for nSES, and for the Pittsburgh sample, railway density. Separate models were fit for both the overall CHCS sample and for the Pittsburgh sample. For the Pittsburgh sample, we also fit separate models for both tract‐level and 1 km buffer level water density to test for differences by spatial unit. We repeated the above modeling sequence for each dementia subtype (Alzheimer's, mixed/vascular). Mixed and vascular cases were combined due to the low number of vascular cases in CHCS, as done previously.^2^, ^37^ Finally, we conducted a sensitivity analysis exploring whether the risk association with dementia differed between industrialized versus non‐industrialized blue spaces. We did this by adding an interaction term between 1 km buffer water density and presence of railways (yes/no) to the above models.

Variance estimates for all models were clustered on baseline Census tract to account for the multilevel data structure. We performed Grambsch–Therneau tests on the fully adjusted models to assess for violations in the proportional hazards assumption. There were no violations of the proportional hazards assumption detected for the models in Pittsburgh (global test *p* = 0.326). In models of the full CHCS sample, the proportional hazards assumption was violated for the independent variable lifetime occupation (global test *p* = 0.064), so we also adjusted for the interaction of lifetime occupation with follow‐up time in these models (see Supplementary Material).

## 3 RESULTS

### 3.1 Participant characteristics

Participants at the Pittsburgh site were age 75 (SD = 4.7) years on average and mostly women (55%), and 19% were Black or African American (Table 1). Most participants had greater than a high school education (55%) and a professional occupation (41%). Most participants had at least one health condition (79%) and reported mild to moderate depressive symptoms, on average (CES‐D score: mean = 5.8, SD = 5.0). Participant characteristics for the overall CHCS sample are reported in Table S1. Participants at the Pittsburgh site were more likely to be Black or African American, have higher income and education, and have had a professional occupation, and reported higher depressive symptoms compared to the remaining CHCS sample (Table S2).

**TABLE 1:** Participant characteristics by neighborhood blue space group in Allegheny County, PA (*N* = 651).

			Neighborhood blue space (proportion of 1 km buffer)			
	Overall		Low (0)	Middle (>0–0.002)	High (>0.002)	
Variable	M ± SD or *N* (%)	Range	M ± SD or *N* (%)	M ± SD or *N* (%)	M ± SD or *N* (%)	*p*‐value
Age	75.1 ± 4.7	66–91	75.2 ± 4.7	73.9 ± 5.2	75.1 ± 4.4	0.268
Woman (vs man)	358 (55%)		303 (55%)	20 (54%)	35 (56%)	0.989
Black/African American (vs White)	126 (19%)		108 (20%)	7 (19%)	11 (17%)	0.918
Income						0.001
[12K)	130 (20%)		101 (18%)	6 (16%)	23 (37%)	
[12–25K)	190 (29%)		158 (29%)	8 (22%)	24 (38%)	
[25–35K)	67 (10%)		62 (11%)	2 (5%)	3 (5%)	
[>35K]	210 (32%)		184 (33%)	18 (49%)	8 (13%)	
Education						<0.001
< High school	102 (16%)		83 (15%)	0 (0%)	19 (30%)	
High school degree/GED	191 (29%)		168 (30%)	8 (22%)	15 (24%)	
> High school	358 (55%)		300 (54%)	29 (78%)	29 (46%)	
Occupation						0.030
Professional	268 (41%)		236 (43%)	17 (46%)	15 (24%)	
Service	102 (16%)		88 (16%)	4 (11%)	10 (16%)	
Laborer	78 (12%)		62 (11%)	3 (8%)	13 (21%)	
Housewife	138 (21%)		117 (21%)	8 (22%)	13 (21%)	
Other	65 (10%)		48 (9%)	5 (14%)	12 (19%)	
Comorbidity index						0.025
0	197 (30%)		166 (30%)	18 (49%)	13 (21%)	
1	296 (45%)		252 (46%)	15 (41%)	29 (46%)	
≥2	158 (24%)		133 (24%)	4 (11%)	21 (33%)	
Depressive symptoms (CES‐D)	5.8 ± 5.0	0–28	5.7 ± 5.0	5.0 ± 4.9	6.7 ± 5.4	0.222
Physical activity (kcal/week)	1387.1 ± 1639.5	0–11104	1352.3 ± 1617.1	1609.3 ± 1686.7	1561.1 ± 1804.9	0.442

*Note*: *n* = 651 from Allegheny County, PA (Pittsburgh study site) were included. Neighborhood blue space groups were stratified using approximate tertiles of water density (km^2^) within a 1 km radial buffer around the home address. *p*‐values are from analyses of variance (ANOVAs) of continuous variables and chi‐square tests of categorical variables.

Abbreviations: CES‐D, Centers for Epidemiological Studies Depression Scale; kcal, kilocalories; km, kilometer; M, mean; SD, standard deviation.

Baseline covariates differed by blue space tertile in the overall sample (Table S1). Participants in areas with high blue space density tended to have lower income and education (*p* < 0.05 for all). These socioeconomic differences were similar yet stronger in magnitude for the Pittsburgh site (Table 1). For example, participants in Pittsburgh with higher blue space were also less likely to have had a professional occupation and reported a higher number of medical conditions (Table 1). There were no differences in age, sex, or race by blue space tertile at the Pittsburgh site.

There were 464 incident dementia cases (14.8%) in the analytic sample, of which 236 (50.9%) were classified as Alzheimer's disease, 59 (12.7%) as vascular, 148 (31.9%) as mixed Alzheimer's/vascular, and 21 (4.5%) as other pathologies (e.g., Lewy body). There were 102 incident cases (14.7%) in the Pittsburgh subsample (Alzheimer's: *n* = 53, 52.0%; vascular: *n* = 16, 15.7%; mixed: *n* = 27, 26.5%; other: *n* = 6, 5.9%).

### 3.2 Neighborhood characteristics

**FIGURE 1:**
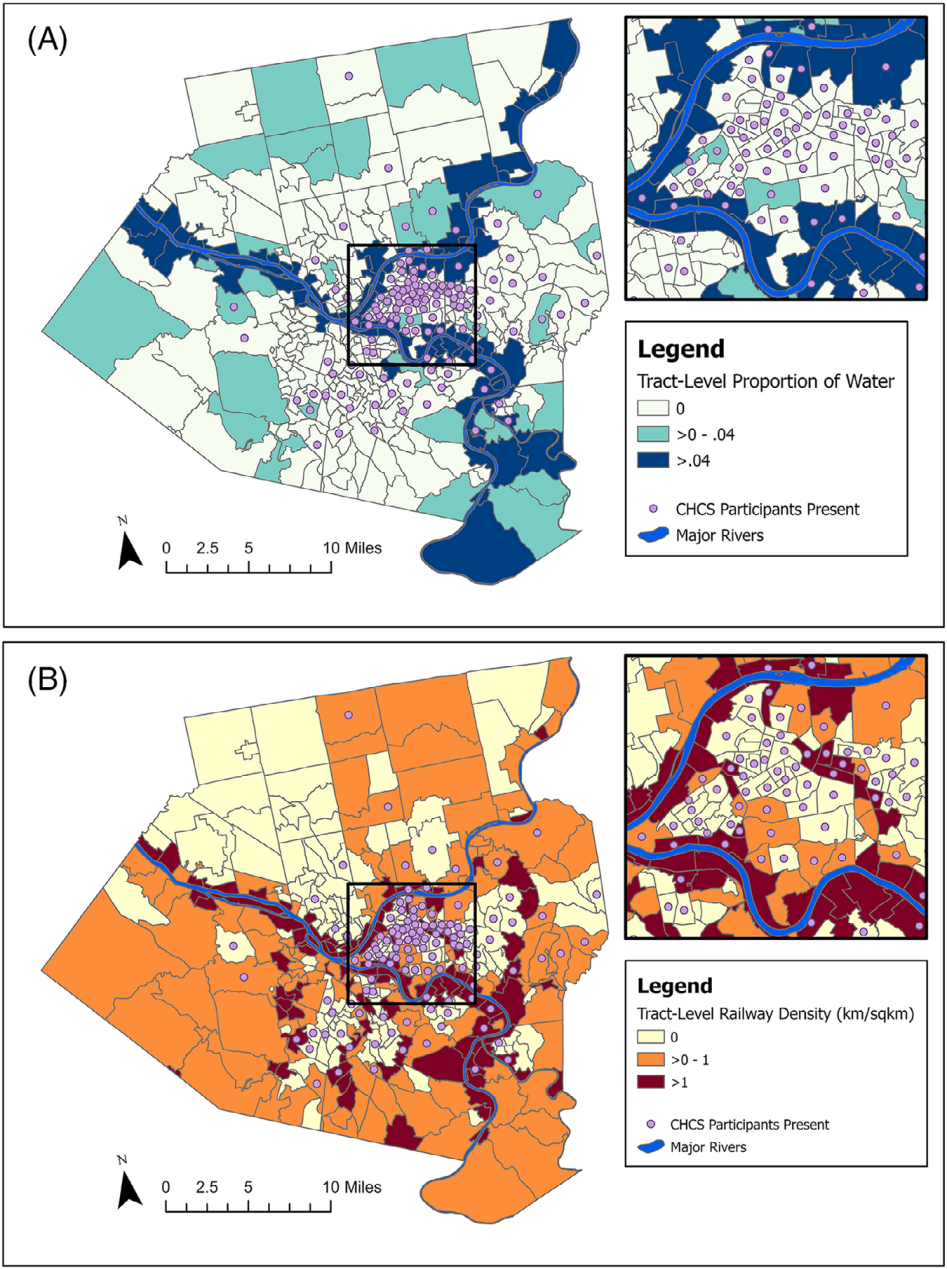
Spatial overlap of blue space (A) and industrial railway density (B) in Allegheny County, PA.

Tract‐level proportions of open water and the major rivers in Allegheny County are mapped in Figure 1A. Tracts with the highest proportion of water were mostly adjacent to major rivers, and most tracts within Allegheny County had no water. Several tracts with high proportions of water also had high railway density (Figure 1B). Greater buffer‐level water density was also correlated with greater railway density (*r* = 0.47) and lower nSES (*r* = ‐0.27) at the Pittsburgh site (Table S3).

### 3.3 Neighborhood blue space and risk of dementia

**FIGURE 2:**
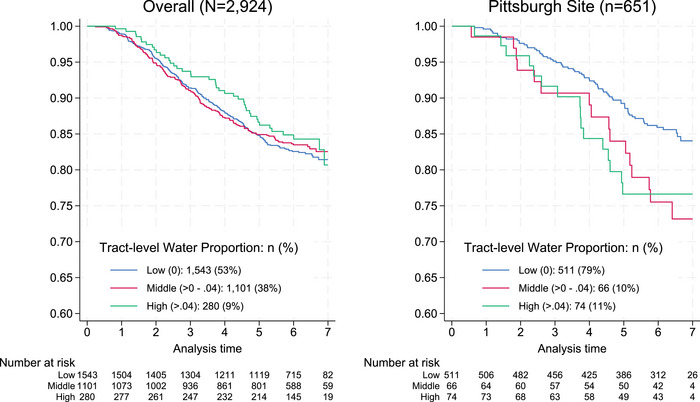
Incident risk of all‐cause dementia by tract‐level blue space in the Cardiovascular Health Cognition Study. Note. Plots were truncated at Year 7 due to low remaining sample size at risk.

At the Pittsburgh site, higher tract‐level water density was associated with a higher risk of incident dementia (Figure 2). The highest tertile of tract‐level water density was associated with 2.03 times the hazard rate of dementia (Table 2, Model 2, hazard ratio [HR] = 2.03, 95% confidence interval [CI]: 1.40–2.93, *p* < 0.001), after adjusting for individual demographic, health, and socioeconomic covariates. This association was attenuated and no longer statistically significant after further adjusting for nSES and railway density (Model 3—HR = 1.61, 95% CI: 0.97–2.67, *p* = 0.064).

**TABLE 2:** Adjusted associations between neighborhood blue space and dementia risk in Allegheny County, PA (*N* = 651).

	Model 1^*^		Model 2^†^		Model 3^‡^	
	HR (95% CI)	*p*‐value	HR (95% CI)	*p*‐value	HR (95% CI)	*p*‐value
Tract‐level water						
Low (0) (ref.)						
Middle (>0–0.04)	1.89 (1.38–2.60)	<0.001	2.01 (1.35–3.00)	0.001	1.79 (1.13–2.84)	0.013
High (>0.04–0.22)	2.04 (1.42–2.92)	<0.001	2.03 (1.40–2.93)	<0.001	1.61 (0.97–2.67)	0.064
1‐km buffer water						
Low (0) (ref.)						
Middle (>0–0.002)	0.87 (0.40–1.86)	0.713	1.06 (0.42–2.70)	0.902	0.90 (0.36–2.24)	0.823
High (>0.002–0.16)	1.82 (1.07–3.12)	0.028	1.65 (0.92–2.96)	0.094	1.29 (0.66–2.55)	0.455

*Note*: Blue space measures included the proportion of open water at the tract level and 1‐km buffer level, which were modeled separately. Estimates are hazard ratios (HRs) comparing risk of incident dementia in the higher neighborhood blue space tertiles to the lowest tertile.

* Model 1 was age‐adjusted.

^†^ Model 2 further adjusted for gender (man, woman), race (Black/African‐American, White), individual education (< high school, high school degree/GED, > high school), income (<$12k, [$12–25k), [$25–35k), ≥$35k, missing), lifetime occupation (professional, service, laborer, housewife, other), physical activity (kcal/week), depressive symptoms (CES‐D score), and number of medical conditions (cardiovascular disease, cerebrovascular disease, diabetes, hypertension).

^‡^ Model 3 further adjusted for tract‐level neighborhood socioeconomic status (nSES) and tract‐level railway density.

Associations for the Pittsburgh site were attenuated when using 1 km radial buffers versus tract‐level measures of blue space density (Table 2). The highest tertile of buffer‐level water density was associated with 1.82 times the hazard rate of dementia in the age‐adjusted model (Model 1—HR = 1.82, 95% CI: 1.07–3.12, *p* = 0.028). This association was no longer significant after adjusting for individual demographic and socioeconomic covariates (Model 2—HR = 1.65, 95% CI: 0.92–2.96, *p* = 0.094) and was further attenuated after adjusting for neighborhood confounders, including nSES and railway density (Model 3–HR = 1.29, 95% CI: 0.66–2.55, *p* = 0.455). We found no significant association between water density and incident dementia in the fully adjusted model for the overall CHCS sample (Table S4).

Sensitivity analyses suggested that the risk association differed by dementia subtype. The hazard rate of mixed/vascular dementia was 2.77 times higher for the highest tertile of water density, adjusting for individual demographic, health, and socioeconomic covariates (Table 3; Model 2—HR = 2.77, 95% CI: 1.14–6.76, *p* = 0.025). This association was modestly attenuated after further adjusting for nSES and railway density (Model 3—HR = 2.65, 95% CI: 0.99–7.07, *p* = 0.052). There were no significant associations between water density and incident Alzheimer's disease (*p* > 0.05, Table 3).

**TABLE 3:** Adjusted associations between neighborhood blue space and dementia subtypes in Allegheny County, PA (*N* = 651).

	Model 1^*^		Model 2^†^		Model 3^‡^	
	HR (95% CI)	*p*‐value	HR (95% CI)	*p*‐value	HR (95% CI)	*p*‐value
**Alzheimer's subtype** 1‐km buffer water						
T1 (0) (ref.)						
T2 (>0–0.002)	0.79 (0.27–2.30)	0.670	0.84 (0.24–2.90)	0.783	0.69 (0.22–2.13)	0.512
T3 (>0.002–0.16)	1.33 (0.70–2.54)	0.384	1.15 (0.55–2.42)	0.713	0.74 (0.33–1.64)	0.454
**Mixed/vascular subtype** 1‐km buffer water						
T1 (0) (ref.)						
T2 (>0–0.002)	1.11 (0.40–3.09)	0.836	1.95 (0.64–5.93)	0.237	1.77 (0.56–5.62)	0.334
T3 (>0.002–0.16)	2.87 (1.43–5.74)	0.003	2.77 (1.14–6.76)	0.025	2.65 (0.99–7.07)	0.052

*Note*: Blue space measures included the proportion of open water at the 1 km buffer level. Estimates are hazard ratios (HRs) comparing the risk of incident dementia in the higher neighborhood blue space tertiles to the lowest tertile.

* Model 1 was age‐adjusted.

^†^ Model 2 further adjusted for gender (man, woman), race (Black/African‐American, White), individual education (< high school, high school degree/GED, > high school), income (<$12k, [$12–25k), [$25–35k), ≥$35k, missing), lifetime occupation (professional, service, laborer, housewife, other), physical activity (kcal/week), depressive symptoms (CES‐D score), and number of medical conditions (cardiovascular disease, cerebrovascular disease, diabetes, hypertension).

^‡^ Model 3 further adjusted for tract‐level neighborhood socioeconomic status (nSES) and tract‐level railway density.

To examine industrialized versus non‐industrialized blue spaces, we cross‐tabulated 1 km buffer water density tertiles by presence of railways (Table S5). Notably, very few participants (8%) lived in areas with blue space but without railways (Table S5). There was no significant interaction between buffer‐level water density and presence of railways in adjusted models (Table S6, *p* = 0.397). In exploratory stratified analyses, there was no association between buffer‐level water density and dementia in areas without railways (Table S6, *p* > 0.05 for all). Yet, in areas with railways, the highest tertile of buffer‐level water density was associated with 2.57 times the hazard rate of dementia after full adjustment (Table S6; Model 3—HR = 2.57, 95% CI: 1.02–6.48, *p* = 0.045).

## 4 DISCUSSION

Here we expanded on limited existing research on blue spaces as a potential risk factor for incident ADRD in community‐dwelling older adults. Of note, we demonstrated that this observed relationship varied based on the spatial unit used to measure blue space and after accounting for historical social confounders. We found no association between blue space density and incident ADRD in the overall CHCS sample. Yet, at the Pittsburgh site, we found that tract‐level blue space density was associated with increased risk of ADRD, and specifically the mixed/vascular subtype. This association was attenuated when using 1 km radial buffers to more precisely quantify nearby blue space. It was further attenuated after adjusting for individual and neighborhood factors important for contextualizing major blue spaces in Pittsburgh. Our results illustrate the importance of accounting for spatial, historical, and social contexts when examining associations between natural environmental features and cognitive aging outcomes, particularly in local settings.

Our findings build upon recent studies examining links between blue spaces and clinical cognitive outcomes. Wu and colleagues found that the percentage of blue space cover within a 400 or 800 m radius of participant home addresses was not associated with an increased odds of dementia diagnosis in a cross‐sectional analysis of two national cohort studies.^7^ In a study of U.S. Medicare beneficiaries, Klompmaker and colleagues found that percentage of blue space cover at the zip code‐level was associated with significant decrease in hospitalizations from Parkinson's disease, but not from dementia.^6^ Notably, these studies did not further examine differences by local geographic contexts, spatial unit (tract vs buffer), or by dementia subtypes.

Here we found that the observed associations between blue space density and incident dementia were stronger for tract‐level versus buffer‐level measures. Ideally, selected spatial units should reflect the spatial scale at which environmental exposures influence a health outcome. The buffer measures were designed to better capture blue spaces within immediate walking distance of the home, thus more closely approximating individual‐level exposure.^17^ Buffers also tend to capture spatial heterogeneity across participants, even those living within the same tract, potentially improving measurement precision and reducing exposure misclassification.

Paradoxically, however, the tract‐level associations were stronger. This may reflect several phenomena. First, tract‐level measures may align more closely with broader contextual or ecological processes (e.g., neighborhood investment in waterfront development, regional zoning patterns) that influence dementia risk via more distal or community‐level mechanisms.^27^ Second, buffer‐based estimates, despite being more granular, are vulnerable to spatial uncertainty, particularly from address geocoding error, boundary misalignment, or day‐to‐day activity space variation, which could introduce non‐differential measurement error and attenuate observed associations.^38^, ^39^ In contrast, tract‐level measures, although coarser, may provide more stable and less error‐prone proxies of neighborhood context.^40^, ^41^ These findings highlight the modifiable areal unit problem (MAUP), where the observed associations can vary depending on the spatial unit of analysis.^42^, ^43^ They underscore the importance of conducting sensitivity analyses using multiple spatial scales, and exercising caution when relying solely on administrative boundaries (e.g., census tracts, ZIP Code tabulation areas) to quantify environmental exposures. Ultimately, aligning the spatial scale of measurement with the hypothesized scale of effect, while accounting for sources of spatial error, is critical in environmental epidemiology.^44^, ^45^

Our findings also highlight the key role of the broader neighborhood social context in characterizing the risk of blue spaces on cognitive health. We observed marked differences in estimates when comparing the overall CHCS sample to the local Pittsburgh site, where neighborhoods with major blue spaces were historically industrialized and working‐class.^18^, ^19^ We found that associations for the Pittsburgh sample were strongly attenuated after adjusting for neighborhood‐level socioeconomic resources and railway density (i.e., proxy measure of industrialization). Emerging literature suggests that lower neighborhood socioeconomic status may increase risk of dementia via diverse mechanisms, including quantity and quality of amenities to maintain healthy behaviors.^2^, ^27^, ^46^ In addition to their role as independent confounders, industrialization and socioeconomic conditions may also influence the usability and accessibility of blue spaces. In Pittsburgh, major rivers were historically used for industrial shipping rather than recreation, especially during the period when CHCS participants were emerging adults.^18^, ^19^ Individuals in urban neighborhoods with lower socioeconomic status have also been shown to have lower access to blue spaces and parks promoting recreation.^47^

Examination of dementia subtypes provided further insight into the pathological pathways through which blue spaces may impact ADRD. Here we found that blue space was associated with a greater risk of mixed/vascular dementia, but not Alzheimer's disease. Because cases classified as Alzheimer's disease in CHCS lacked significant vascular pathology (e.g., cerebral infarcts, white matter lesions), this may suggest that blue spaces influence dementia risk primarily through a vascular pathway. Industrialized blue spaces may impact cardiovascular health via increased stress and chronic inflammation (e.g., elevated inflammatory cytokines) from surrounding noise and air pollution.^16^, ^20^, ^48^ Exposure to air pollution has also been linked with greater cardiorespiratory pathology (e.g., hypertension, arterial stiffening, high‐density lipoprotein dysfunction) and increased risk of stroke and atherosclerosis, both of which are key contributors to vascular dementia.^21^ Blue spaces used primarily for industrial purposes may further lack opportunities for recreational physical activity, and some studies have reported that physical activity is more strongly related to vascular dementia than to Alzheimer's disease.^49^, ^50^, ^51^ We also previously found in CHCS that having fewer neighborhood resources promoting physical activity was associated with a higher risk of cardiovascular disease and mixed/vascular dementia, but not Alzheimer's disease, providing additional support for a potential physical activity‐related vascular pathway.^2^, ^52^

It is notable that our study included primarily blue spaces from historically industrialized and working‐class neighborhoods of Pittsburgh, and it is plausible that more recreational blue spaces with less industrial activity may instead strengthen cardiovascular health and benefit cognitive functioning.^9^, ^10^, ^11^ Future studies with broader spatial representation should therefore move beyond singular measures of blue space quantity and further contextualize different types of blue spaces (e.g., recreational vs industrialized) to assess their differential effects on cognitive aging. We began to explore this in the current study, finding that dementia risk was higher, specifically in more industrialized blue spaces with railways present. This provides preliminary support that the uses and surrounding context of blue spaces may dictate their effects on cognitive aging. Yet, interpreting these findings warrants caution, given that the interaction term in our model was not statistically significant, and that railway presence is an imperfect proxy for industrialization. We also lacked sufficient spatial representation of areas without industrialized blue spaces, which may in the future be addressed through including populations from the more rural surrounding areas of Allegheny County.

### 4.1 Limitations and strengths

Additional limitations of this study included our lack of residential histories of participants prior to study enrollment. This prevented us from assessing residential mobility prior to study enrollment and accounting for length of exposure to blue spaces earlier in the lifespan. We adjusted for individual education and lifetime occupation, but we lacked measures of early childhood socioeconomic conditions (e.g., parental education) that may further confound the relationship between lifespan neighborhood exposures and dementia risk.^53^ Radial buffer measures, despite having increased precision over tract‐level measures, are also dependent on specific assumptions (e.g., selection of buffer size) and are unable to capture actual use of blue spaces. Future work can utilize road network buffers^54^ or location‐based tracking (e.g., wearable global positioning system [GPS] units)^55^ to more precisely capture accessible blue spaces.

Our study also had several key strengths. CHCS is a large, biracial cohort with clinically adjudicated dementia diagnoses from multiple data sources. We also used a prospective study design, providing stronger support for the temporal precedence of blue space exposure on incident dementia compared to prior cross‐sectional studies. We further considered multiple individual‐ and neighborhood‐level confounders and evaluated the consistency of our findings across different spatial units.

## 5 CONCLUSION

Our findings underscore the complexity of evaluating how blue spaces and other natural environmental features influence cognitive health in aging populations. Although we observed no consistent evidence that blue space density was associated with all‐cause dementia, we found that it predicted mixed/vascular dementia. Our results further suggest that any potential relationship with blue space may vary across spatial, historical, and social contexts. Accounting for these contextual factors involves careful selection of spatial units and covariate measures to account for potential confounding, which can be further informed by local communities familiar with the area's social context and history. To meaningfully build upon this literature, we recommend that future studies (1) better distinguish types of blue spaces (e.g., recreational vs non‐recreational) using additional historical and contemporary environmental measures (e.g., industrial density), and (2) incorporate sensitivity analyses to assess the robustness of findings across spatial scales. To inform effective interventions, future work must consider how environmental influences on cognitive aging vary by place and population.

## CONFLICT OF INTEREST STATEMENT

The authors declare no conflicts of interest. Any author disclosures are available in the Supporting Information.

## CONSENT STATEMENT

All human subjects provided written informed consent.

## Supporting information



Supporting Information

Supporting Information
