# Supplementary material for: Blue spaces and incident dementia: Differences by geospatial and historical contexts
Source: Alzheimers Dement. 2025 Oct 28;21(10):e70850. doi: 10.1002/alz.70850 (PMC12568378; doi:10.1002/alz.70850)
Supplement: Supplementary file 1 — Supporting Information [file ALZ-21-e70850-s001.docx]

**Blue spaces and incident dementia: Differences by geospatial and historical contexts**

**Supplemental Appendix**

Table S1. Participant Characteristics by Neighborhood Blue Space Group in the Cardiovascular Health Cognition Study

Table S2. Participant Characteristics for Pittsburgh Study Site Versus the Remaining Cardiovascular Health Cognition Study Sample

Table S3. Spearman Correlations between Neighborhood Measures at Pittsburgh Site

Table S4. Adjusted Associations between Neighborhood Blue Space and Dementia Risk for Overall Sample in the Cardiovascular Health Cognition Study

Table S5: Cross-tabulation of Blue Space Tertiles by Presence of Railways in Pittsburgh

Table S6: Adjusted Associations of Blue Space on Dementia Risk in Pittsburgh, Stratified by Railroad Density

**Table S1. Participant Characteristics by Neighborhood Blue Space Group in the Cardiovascular Health Cognition Study**

|  |  |  | **Neighborhood Blue Space (1km buffer)** | | |  |
| --- | --- | --- | --- | --- | --- | --- |
| **Variable** | **Overall** |  | **Low (0)** | **Middle (>0-.002)** | **High (>.002)** |  |
|  | **M±SD or N (%)** | **Range** | **M±SD or N (%)** | **M±SD or N (%)** | **M±SD or N (%)** | **P-value** |
| Age | 75.0±4.9 | 64-98 | 75.0±5.0 | 74.7±4.7 | 75.3±4.8 | 0.087 |
| Woman (vs. man) | 1684 (58%) |  | 859 (57%) | 398 (59%) | 427 (57%) | 0.853 |
| Black/African-American (vs. White) | 414 (14%) |  | 293 (20%) | 69 (10%) | 52 (7%) | <.001 |
|  |  |  |  |  |  |  |
| Income |  |  |  |  |  | 0.008 |
| *[12K)* | 625 (21%) |  | 291 (19%) | 167 (25%) | 167 (22%) |  |
| *[12,25K)* | 1000 (34%) |  | 499 (33%) | 225 (33%) | 276 (37%) |  |
| *[25,35K)* | 440 (15%) |  | 226 (15%) | 98 (14%) | 116 (16%) |  |
| *[>35K]* | 688 (24%) |  | 379 (25%) | 158 (23%) | 151 (20%) |  |
|  |  |  |  |  |  |  |
| Education |  |  |  |  |  | <.001 |
| *<High school* | 717 (25%) |  | 305 (20%) | 189 (28%) | 223 (30%) |  |
| *High school degree/GED* | 840 (29%) |  | 426 (28%) | 193 (28%) | 221 (30%) |  |
| *>High school* | 1367 (47%) |  | 768 (51%) | 298 (44%) | 301 (40%) |  |
|  |  |  |  |  |  |  |
| Occupation |  |  |  |  |  | 0.011 |
| *Professional* | 1102 (38%) |  | 592 (39%) | 250 (37%) | 260 (35%) |  |
| *Service* | 430 (15%) |  | 247 (16%) | 89 (13%) | 94 (13%) |  |
| *Laborer* | 473 (16%) |  | 220 (15%) | 114 (17%) | 139 (19%) |  |
| *Housewife* | 633 (22%) |  | 299 (20%) | 162 (24%) | 172 (23%) |  |
| *Other* | 286 (10%) |  | 141 (9%) | 65 (10%) | 80 (11%) |  |
|  |  |  |  |  |  |  |
| Comorbidity Index |  |  |  |  |  | 0.651 |
| *0* | 793 (27%) |  | 418 (28%) | 189 (28%) | 186 (25%) |  |
| *1* | 1377 (47%) |  | 695 (46%) | 318 (47%) | 364 (49%) |  |
| *≥2* | 754 (26%) |  | 386 (26%) | 173 (25%) | 195 (26%) |  |
| Depressive symptoms (CES-D) | 5.1±4.7 | 0-29 | 5.3±4.9 | 4.7±4.4 | 5.2±4.6 | 0.035 |
| Physical activity (kcal/week) | 1477.5±1758.4 | 0-13251 | 1433.4±1722.9 | 1465.8±1766.4 | 1577.1±1819.1 | 0.186 |

Note. N=2,924 participants were included. Neighborhood blue space groups were stratified using approximate tertiles of water density (km^2^) within a 1-km radial buffer around the home address. P-values are from ANOVAs of continuous variables and chi-square tests of categorical variables.

**Table S2. Participant Characteristics for Pittsburgh Study Site Versus the Remaining Cardiovascular Health Cognition Study Sample**

| **Variable** | **Pittsburgh Site**  **(n=651)** | **Remaining CHCS Sample (n=2,273)** |  |
| --- | --- | --- | --- |
|  | **M±SD or N (%)** | **M±SD or N (%)** | **P-value** |
| Age | 75.1±4.7 | 75.0±5.0 | 0.605 |
| Women (vs. men) | 358 (55%) | 1326 (58%) | 0.128 |
| Black/African-American (vs. White) | 126 (19%) | 288 (13%) | <.001 |
|  |  |  |  |
| Income |  |  | <.001 |
| *[12K)* | 130 (20%) | 495 (22%) |  |
| *[12,25K)* | 190 (29%) | 810 (36%) |  |
| *[25,35K)* | 67 (10%) | 373 (16%) |  |
| *[>35K]* | 210 (32%) | 478 (21%) |  |
|  |  |  |  |
| Education |  |  | <.001 |
| *<High school* | 102 (16%) | 615 (27%) |  |
| *High school degree/GED* | 191 (29%) | 649 (29%) |  |
| *>High school* | 358 (55%) | 1009 (44%) |  |
|  |  |  |  |
| Occupation |  |  | 0.014 |
| *Professional* | 268 (41%) | 834 (37%) |  |
| *Service* | 102 (16%) | 328 (14%) |  |
| *Laborer* | 78 (12%) | 395 (17%) |  |
| *Housewife* | 138 (21%) | 495 (22%) |  |
| *Other* | 65 (10%) | 221 (10%) |  |
|  |  |  |  |
| Comorbidity Index |  |  | 0.119 |
| *0* | 197 (30%) | 596 (26%) |  |
| *1* | 296 (45%) | 1081 (48%) |  |
| *≥2* | 158 (24%) | 596 (26%) |  |
|  |  |  |  |
| Depressive symptoms (CES-D) | 5.8±5.0 | 5.0±4.6 | <.001 |
| Physical activity (kcal/week) | 1387.1±1639.5 | 1503.4±1790.5 | 0.137 |

Note. P-values are from test tests of continuous variables and chi-square tests of categorical variables. CHCS = Cardiovascular Health Cognition Study.

**Table S3. Pearson Correlations between Neighborhood Measures at Pittsburgh Site**

|  | **Water Density** | **Railway Density** |
| --- | --- | --- |
| **Water Density** (1km buffer) | -- |  |
| **Railway Density** | 0.47*** | -- |
| **Neighborhood socioeconomic status (nSES)** | -0.27*** | -0.17*** |

Note. N=651, p<.001***

**Table S4. Adjusted Associations between Neighborhood Blue Space and Dementia Risk for Overall Sample in the Cardiovascular Health Cognition Study**

|  | **Model 1** |  | **Model 2** | | **Model 3** | |
| --- | --- | --- | --- | --- | --- | --- |
|  | **HR (95% CI)** | **p-value** | **HR (95% CI)** | **p-value** | **HR (95% CI)** | **p-value** |
| **Proportion Open Water (1-km Buffer)** |  |  |  |  |  |  |
| T1 (0) (ref.) |  |  |  |  |  |  |
| T2 (>0 - .002) | 1.20 (0.95, 1.50) | 0.130 | 1.29 (1.01, 1.64) | 0.039 | 1.27 (0.98, 1.63) | 0.066 |
| T3 (>.002 - .16) | 1.07 (0.86, 1.34) | 0.534 | 1.01 (0.80, 1.28) | 0.933 | 1.00 (0.79, 1.27) | 0.993 |

Note. N=2,924. Model 1 was unadjusted. Model 2 was adjusted for individual age, sex, race, education, income, lifetime occupation, physical activity expenditure, mobility limitation (reported difficulty walking a half mile), physical activity (kcal/week), depressive symptoms (CES-D score), and number of medical conditions (cardiovascular disease, cerebrovascular disease, diabetes, hypertension). Model 3 was further adjusted for neighborhood socioeconomic status (nSES). The proportional hazards assumption was violated after adjusting for lifetime occupation, so interactions between lifetime occupation and follow-up time were included in Models 2 and 3.

**Table S5: Cross-tabulation of Blue Space Tertiles by Presence of Railways in Pittsburgh**

|  | Railways |  |  |  |
| --- | --- | --- | --- | --- |
|  | No | Yes |  | Total |
| 1-km Buffer Water Density | N (%) | N (%) |  | N (%) |
| T1 (0) (ref.) | 332 (92) | 219 (75) |  | 551 (84) |
| T2 (>0 - .006) | 12 (3) | 25 (9) |  | 37 (6) |
| T3 (>.006 - .49) | 17 (5) | 46 (16) |  | 63 (10) |
| Total | 361 (100) | 290 (100) |  | 651 (100) |

Note. Column percentages are reported.

**Table S6: Adjusted Associations of Blue Space on Dementia Risk in Pittsburgh, Stratified by Railroad Density**

|  | No Railways (n=361) | Any Railways (n=290) | P-value (interaction) |
| --- | --- | --- | --- |
| 1-km Buffer Water Density | HR (95% CI) | HR (95% CI) |  |
| T1 (0) (ref.) |  |  | 0.397 |
| T2 (>0 - .006) | 1.02 (0.09, 12.12) | 0.78 (0.23, 2.63) |  |
| T3 (>.006 - .49) | 0.71 (0.21, 2.36) | 2.57 (1.02, 6.48)* |  |

Note. Estimates are hazard ratios comparing risk of incident dementia in the higher neighborhood blue space tertiles to the lowest tertile. Models were adjusted for age, gender, race, study site, individual education, lifetime occupation, physical activity, depressive symptoms, number of medical conditions, and tract-level neighborhood socioeconomic status (nSES). Moderation was tested by including an interaction term between water density tertile and presence of railways, and the stratified results are presented. *p<.05
